# Substance Use, Homelessness, Mental Illness and Medicaid Coverage: A Set-up for High Emergency Department Utilization

**DOI:** 10.5811/westjem.2018.9.38954

**Published:** 2018-10-18

**Authors:** Aimee Moulin, Ethan J. Evans, Guibo Xing, Joy Melnikow

**Affiliations:** * University of California, Davis, Department of Emergency Medicine, Department of Psychiatry, Davis, California; † University of California, Davis, Center for Healthcare Policy and Research, Davis, California; ‡ University of California, Davis, Department of Family and Community Medicine, Davis, California

## Abstract

**Introduction:**

Frequent users of emergency departments (ED) account for 21–28% of all ED visits nationwide. The objective of our study was to identify characteristics unique to patients with psychiatric illness who are frequent ED users for mental health care. Understanding unique features of this population could lead to better care and lower healthcare costs.

**Methods:**

This retrospective analysis of adult ED visits for mental healthcare from all acute care hospitals in California from 2009–2014 used patient-level data from California’s Office of Statewide Health Planning and Development. We calculated patient demographic and visit characteristics for patients with a primary diagnosis of a mental health disorder as a percentage of total adult ED visits. Frequent ED users were defined as patients with more than four visits in a 12-month period. We calculated adjusted rate ratios (aRR) to assess the association between classification as an ED frequent user and patient age, sex, payer, homelessness, and substance use disorder.

**Results:**

In the study period, 846,867 ED visits for mental healthcare occurred including 238,892 (28.2%) visits by frequent users. Patients with a primary mental health diagnosis and a co-occurring substance use diagnosis in the prior 12 months (77% vs. 37%, aRR [4.02], 95% confidence interval [CI] [3.92–4.12]), homelessness (2.9% vs 1.1%, odds ratio [1.35], 95% [CI] [1.27–1.43]) were more likely to be frequent users. Those covered by Medicare (aRR [3.37], 95% CI [3.20–3.55]) or the state’s Medicaid program Medi-Cal (aRR [3.10], 95% CI [2.94–3.25]) were also more likely to be frequent users compared with those with private insurance coverage.

**Conclusion:**

Patients with substance use disorders, homelessness and public healthcare coverage are more likely to be frequent users of EDs for mental illness. Substance use and housing needs are important factors to address in this population.

## INTRODUCTION

Mental illness is widespread and has high medical and socioeconomic costs.^1^–^5^ Emergency department (ED) visits for mental healthcare are growing in the United States (U.S.).^6^,^7^ Many patients continue to face significant barriers to consistent mental healthcare.^2^,^8^–^11^ ED visits increase when mental health services are unavailable or uncoordinated.^12^–^14^ Nationally, frequent ED users for all diagnoses account for 3–8% of all ED patients and 21–28% of all ED visits.^15^–^17^ High ED utilization is often seen as a marker of unmet healthcare needs as well as an opportunity to decrease healthcare costs and improve resource utilization.^15^,^18^,^19^ Yet prior research on frequent ED users found that these patients have multiple chronic conditions and high rates of primary and specialty care outside the ED.^17^,^20^ Studies of patients with high ED use for any diagnosis show that they have insurance coverage and are more likely to have private insurance or Medicare insurance.^17^,^20^,^21^

Patients with mental illness face barriers to consistent outpatient care. Mental health services tend to be difficult to access and poorly integrated with primary care.^22^–^24^ Studies on ED utilization in patients with mental illness have focused on large urban populations and may not be generalizable to broader areas. Studies have evaluated ED utilization by patients with mental illness but are limited by the sample being either a single hospital or across a single urban area.^23^,^25^–^27^ A study of ED visits in San Diego by patients with psychiatric diagnosis found that frequent users were more likely to have lower socioeconomic status, homelessness, and co-occurring substance use disorders.^26^

Our study examined ED utilization for patients with a primary mental health diagnosis over a six-year period across California, using data that included the geographic and socioeconomic diversity of the entire state. We hypothesized that patients with mental illness covered by Medicare or Medi-Cal (the state’s Medicaid insurance program), those who were concurrent substance users, and homeless patients would be more likely to have high ED utilization. Understanding factors associated with high ED utilization across a large, diverse state has clinical and policy implications as systems attempt to address ED utilization and healthcare costs.

## METHODS

We conducted a retrospective analysis of all adult ED visits to acute care hospitals with a primary mental illness in California from 2009–2014 using a cohort defined from patient-level data for all ED visits, reported to California’s Office of Statewide Health Planning and Development (OSHPD). Each patient discharged from inpatient admission or ED treatment encounter in a licensed hospital in California is included in the OSHPD data. Our analysis included data on all ED visits from patients discharged or admitted through the ED from 2009–2014. These data do not represent a sample but rather surveillance with 100% coverage. The University of California Davis Institutional Review Board Administration as well as OSHPD’s Committee for the Protections of Human Subjects approved this study.

Data used for the study included a unique patient identification number, patient demographic information to the level of Zip Code, date of service, expected source of payment, disposition, and up to 25 *International Statistical Classification of Diseases and Related Health Problems,* version 9 (ICD-9) diagnosis codes. We defined a surrogate marker for ED encounters of patients with a primary mental illness diagnosis as visits with mental health diagnosis in the first diagnosis position, using ICD-9 codes. Patients with a substance use disorder were defined as patients with a substance use diagnosis using ICD-9 codes in any one of the 24 secondary diagnosis positions. We defined patients with four or more ED encounters for a primary mental illness diagnosis in a 12-month period as frequent ED users. In the OSHPD database patients who were “homeless” were specifically assigned a zip code of “ZZZZZ.” This designation is distinct from patients with an unknown Zip Code reported as “XXXX” and patients who do not reside in the U.S. reported as “YYYY.”

We calculated descriptive analyses of patient demographic and visit characteristics (Table 1). Multivariate log-linear model with Poisson distribution was used to assess the association between patient factors such as age, sex, payer, homelessness, substance use disorder, and classification as an ED frequent user. We used adjusted rate ratios (aRR) to account for variations in person/time using the Poisson log-linear model. aRR and 95% confidence interval (CI) are reported in Table 2. Data analyses were performed using SAS (V9.4) software.

## RESULTS

During the study period, a total of 846,867 visits were made to California EDs by adult patients with mental illness and a valid record linkage number. This total includes patients admitted, transferred, or discharged from the ED. Mean age was 54.0 (standard deviation 21.1) and 55.8% were male. Insurance status was 20.4% Medi-Cal, 31.5 Medicare, 12.4 private insurance, 10.2 % self-pay and 25.5% other (Table 1). Overall 238,892 (28.2%) of ED visits for mental illness were by frequent users.

Frequent users with mental illness had different characteristics than non-frequent users. Patients with a primary mental health diagnosis and a co-occurring, substance use diagnosis in the prior 12 months (77% vs. 37%, aRR [4.02], 95% CI [3.92–4.12]), homelessness (2.9% vs. 1.1%, odds ratio [1.35], 95% CI [1.27–1.43]) were more likely to be frequent users. Those covered by Medicare (aRR [3.37], 95% CI [3.20–3.55]) or Medi-Cal (aRR [3.10], 95% CI [2.94–3.25]) were also more likely to be frequent users compared with those with private insurance coverage.

## DISCUSSION

Frequent ED users are a focus point for many health service agencies and policymakers because of the cost incurred from such patients on healthcare systems. Mental healthcare needs are often identified in the literature as a reason for high ED utilization.^23^,^25^–^27^ However, in many other studies this conclusion is based on including all patients for whom a mental health diagnosis code appears in the case file, i.e., a code in any of the diagnosis lines in a patient file. When a mental health diagnosis from any position is included, mental illness may be a factor in the ED visit but not the primary reason for seeking care. We limited analysis to patients specifically seeking mental health treatment. Using this focused approach we noted several differences between patients who are frequent users of the ED for mental illness and those who are not frequent users, including medical and social conditions that complicate treatment.

In our analysis concurrent, substance use diagnoses had a strong association with frequent ED visits for mental illness. This association between substance use disorders and mental illness highlights the importance of medical treatment that addresses both disorders. According to the Substance Abuse and Mental Health Services Administration’s 2014 National Survey on Drug Use and Health, 7.9 million American adults have co-occurring, substance use disorders and mental illness.^28^ Twenty percent of individuals with a serious mental illness develop a substance use disorder in their lifetime, yet only 7.4% receive treatment for both disorders and 55% receive no treatment at all.^28^ Studies looking at single institutions have found high ED utilization in patients with co-occurring, substance use disorders.^23^,^26^ Such dual-diagnosed patients have low rates of access to treatment for their substance use disorders.^29^ Despite evidence that integrated treatment is considered best practice, there are barriers to widespread adoption.^11^,^30^–^33^ Given the high demand for mental healthcare and substance use treatment identified in this study of California, future research should assess availability and impact of integrated mental health/substance use treatment programs.

Although less strong than the association between co-occurring, substance use disorders, we also found an association between homelessness and frequent ED visits for mental illness. Homeless patients had higher rates of ED visits and hospitalizations than non-homeless patients for all diagnoses, and they reported barriers accessing outpatient care.^34^,^35^ Interventions designed to address homelessness such as supportive housing have shown to impact healthcare utilization and expenditures.^36^–^38^

National databases have shown that Medicaid recipients have a high prevalence of psychiatric disorders,^39^ and psychiatric disorders are a driver of healthcare costs.^40^ Indeed, we found a high proportion of patients entering the ED with mental illness were covered by the state’s Medicaid program Medi-Cal. This finding is consistent with other studies that have noted that patients covered by public insurance are more likely to use the ED when compared with those covered by private insurance.^41^–^43^ Additionally, California extends its Medi-Cal eligibility to the largest extent feasible under federal law. Yet barriers to consistent primary care or lack of access to regular outpatient mental healthcare could explain the higher ED visit rates.^44^,^45^

## LIMITATIONS

Studies that rely on retrospective data can be subject to a set of limitations such as selection, misclassification, and other forms of bias and confounding. Because our data cover the complete, documented population of ED visits in California, selection bias is mitigated. However, this study was dependent on diagnosis codes assigned by the ED provider and was subject to misclassification bias within and across the many hospitals from which patients were included. Further, choosing to identify those visiting the ED for mental health concerns by those with a mental health diagnosis in the first position served only as a proxy and risked missing patients. While individual chart review might have produced less concern, the volume of records made that infeasible. Prior work on ED populations and undiagnosed mental illness suggest that undercounting is more common.^46^ We report on healthcare utilization, but the data cannot speak to health outcomes nor can we definitively identify the causes of high ED utilization. Despite its shortcomings, this study reports and identifies important characteristics of patients who visit EDs for mental illness frequently across a large, diverse population, information that suggests areas for further study.

## CONCLUSION

Patients with substance use diagnoses, patients who are homeless and those who are covered by Medi-Cal, the state’s Medicaid program, are more likely to be frequent users of the ED for mental illness. This suggests substance use and housing needs are important factors to address in patients with high ED use for mental health needs.

## Figures and Tables

**Table 1:** Descriptive statistics for mental health emergency department users.

Patient characteristics	Less than 4 visits/year		4 or more visits/year	
	
	N	%	N	%
Total	607975	71.8	238892	28.2
Gender				
Male	238463	50.1	22592	61.5
Female	237502	49.9	14129	38.5
Age				
21–25	55992	11.8	3916	10.7
26–30	52316	11.0	4922	13.4
31–35	47057	9.9	4700	12.8
36–40	42947	9.0	4123	11.2
41–45	47306	9.9	4493	12.2
46–50	51478	10.8	4815	13.1
51–55	47985	10.1	4277	11.6
56–60	36224	7.6	2752	7.5
61–65	24586	5.2	1512	40.1
66+	70074	14.7	1211	3.3
Payer				
Medi-Cal*	116373	24.4	14795	40.3
Medicare	119080	25.0	10971	29.9
Other	106354	22.3	5001	13.6
Private	54571	11.5	1737	4.7
Self pay	79587	16.7	4217	11.5
Homeless	5079	1.1	1074	2.9
Substance use in past 12 months	176147	37.0	28142	76.6

* Medi-Cal is the Medicaid healthcare program serving low-income people in California.

**Table 2:** Adjusted rate ratio for higher mental health emergency department use.

	Adjusted rate ratio	95% CI
Gender		
Male vs female	1.25	1.22–1.28
Payer		
Medi-Cal vs private	3.10	2.94–3.25
Medicare vs private	3.37	3.20–3.55
Self pay vs private	1.43	1.35–1.51
Other vs private	1.62	1.54–1.71
Age		
20–25 vs 51–55	0.97	0.93–1.01
26–30 vs 51–55	1.13	1.08–1.18
31–35 vs 51–55	1.15	1.10–1.19
36–40 vs 51–55	1.11	1.07–1.16
41–45 vs 51–55	1.08	1.03–1.12
46–50 vs 51–55	1.04	1.00–1.09
56–60 vs 51–55	0.91	0.87–0.96
61–65 vs 51–55	0.81	0.77–0.86
66+ vs 51–55	0.32	0.30–0.35
Homeless	1.35	1.27–1.43
Substance use in past year	4.02	3.92–4.12

*CI*, confidence interval.
